# Limited Changes in Red Blood Cell Parameters After Probiotic Supplementation in Depressive Individuals: Insights from a Secondary Analysis of the PRO-DEMET Randomized Controlled Trial

**DOI:** 10.3390/jcm14010265

**Published:** 2025-01-05

**Authors:** Agata Gajewska, Adam Wysokiński, Dominik Strzelecki, Oliwia Gawlik-Kotelnicka

**Affiliations:** 1 Jonscher Hospital, 93-113 Lodz, Poland; agata.gajewska@stud.umed.lodz.pl; 2 Department of Old Age Psychiatry and Psychotic Disorders, Medical University of Lodz, 92-216 Lodz, Poland; adam.wysokinski@umed.lodz.pl; 3 Department of Affective and Psychotic Disorders, Medical University of Lodz, 92-216 Lodz, Poland; dominik.strzelecki@umed.lodz.pl

**Keywords:** depression, probiotics, microbiota, red blood cells, hematocrit, hemoglobin

## Abstract

**Background**: Depression often coexists with anemia, potentially sharing common pathways, highlighting the need for treatments addressing both conditions simultaneously. This study evaluated the effect of probiotics on red blood cell (RBC) parameters in adults with depressive disorder. We hypothesized that probiotics would positively influence RBC parameters, potentially modulated by baseline inflammation or dietary intake, with improved RBC function correlating with better antidepressant outcomes. **Methods**: This secondary analysis of a two-arm, randomized, double-blind, controlled trial involved 116 adults with depressive disorder. Participants received a probiotic formulation containing *Lactobacillus helveticus Rosell^®^-52* and *Bifidobacterium longum Rosell^®^-175* or a placebo for 60 days. Data from 97 subjects were analyzed for RBC parameters, including hemoglobin (HGB), RBC count, hematocrit (HCT), mean corpuscular volume (MCV), mean hemoglobin concentration (MCH), mean corpuscular hemoglobin concentration (MCHC), and RBC distribution width (RDW). **Results**: Probiotic supplementation did not result in significant changes in RBC parameters compared to the placebo. However, probiotics may help stabilize HGB, HCT, MCH, and MCHC levels, potentially preventing fluctuations observed in the placebo group. **Conclusions**: While probiotics showed potential benefits for depressive symptoms, significant changes in RBC parameters were not observed. Larger studies are needed to clarify the mechanisms and clinical implications.

## 1. Introduction

Depressive disorder and anemia represent distinct medical conditions with complex etiologies, though their co-occurrence is not uncommon and may potentiate individual symptoms [1]. Depressive disorder is a prevalent psychiatric condition characterized by disturbances in mood, cognition, and behavior that significantly impair an individual’s ability to function in life and last for a minimal duration of two weeks [2]. Anemia is a common hematologic disorder characterized by a reduction in hemoglobin (HGB), hematocrit (HCT), or red blood cell (RBC) count, resulting in diminished oxygen-carrying ability in the bloodstream. Clinical symptoms of this illness include weakness, pallor, palpitations, exhaustion, and shortness of breath. Anemia encompasses various types, each marked by distinct changes in RBCs. These alterations are key diagnostic markers and play a critical role in clinical assessment [3]. Interestingly, dysbiosis, characterized by an imbalance in gut microbiota, may play a pivotal role in the above disturbances, leading to various types of anemia by impairing iron absorption, contributing to deficiencies in vitamin B12 or folate, exacerbating chronic diseases, disrupting nutrient uptake, and promoting chronic inflammation [4,5]. The coexistence of depressive disorder with anemia is significant due to a variety of interconnected factors. First, there is a noticeable overlap in symptoms between the two disorders, especially in terms of fatigue, cognitive impairment, and diminished concentration [1]. Furthermore, recent study data suggest a bidirectional association due to shared biological mechanisms between anemia and depression. Inflammatory pathways that have been associated with the onset of depression may also play a role in the development of anemia. Chronic inflammation can alter iron metabolism, which is essential for erythropoiesis—the development of RBCs. Hepcidin, a crucial regulator of iron homeostasis, can be decreased by inflammatory cytokines such as interleukin 6 (IL-6) and tumor necrosis factor alpha (TNF-α).

Hepcidin suppression leads to increased iron sequestration in macrophages and reduced iron availability for erythropoiesis, thereby impairing RBC synthesis [6]. Iron deficiency itself can impact neurotransmitter function and myelination in the nervous system. Iron-dependent enzymes have a role in the synthesis and metabolism of neurotransmitters that impact mood and cognition, such as dopamine, noradrenaline, and serotonin [7]. Iron is crucial for the myelination process because it regulates the expression of genes involved in the development of oligodendrocytes and myelin, as well as the synthesis of essential myelin components including lipids and cholesterol [8].

Moreover, chronic inflammation directly inhibits erythropoiesis by suppressing the proliferation and differentiation of erythroid progenitor cells in the bone marrow. Inflammatory mediators further impair erythropoiesis by interfering with the function of erythropoietin (EPO), a hormone essential for regulating the synthesis of RBCs [9]. Conversely, anemia-related persistent hypoxia has been linked to neurobiological alterations that promote susceptibility to depression. Long-term hypoxia causes changes in the neurotransmitter systems of the brain, especially those involving serotonin, dopamine, and norepinephrine, which are essential for mood regulation [10]. What is more, it may result in structural alterations including synaptic plasticity and neuronal connections in brain areas like the amygdala, hippocampus, and prefrontal cortex that are linked to mood stability [11].

In addition to these physiological factors, co-occurring depression and anemia can significantly lower quality of life and functional ability. Debilitating weariness and decreased motivation, which are hallmarks of both disorders, frequently lead to a vicious cycle in which depression symptoms worsen impairment associated with anemia and vice versa, causing a combined burden on people’s psychosocial functioning [12].

The potential role of probiotics in managing both anemia and depression is an area of growing research interest. A comprehensive understanding of the underlying mechanisms of comorbidities is fundamental for the development of targeted therapies aimed at improving patient outcomes. Probiotics may influence these conditions through various mechanisms. The first important mechanism is the microbiota–gut–brain axis, a bidirectional communication system between the gut and the brain. In depression, changes in gut microbiota composition can cause neurotransmitter imbalances and inflammation, which contribute to mood disorders [13]. Similarly, dysbiosis in anemia can decrease iron absorption and substance metabolism, aggravating deficits [14]. By restoring microbial balance in the gut, probiotics facilitate improved iron absorption in anemia [15] and modulate neurotransmitter levels in depression [16], offering a promising avenue for therapeutic intervention.

Considering inflammation, elevated levels of pro-inflammatory cytokines are linked to both depression and anemia [1]. Probiotics have been shown to have anti-inflammatory qualities by regulating cytokine production and promoting a more balanced immune response. For example, research has shown that probiotic supplementation reduces inflammatory markers including C-reactive protein (CRP) and IL-6 [17]. Additionally, probiotics demonstrate antioxidant properties that help mitigate oxidative stress, a key factor in the pathophysiology of both depression and anemia. Probiotics have the potential to mitigate oxidative stress-induced cellular damage by scavenging reactive oxygen species (ROS) and upregulating antioxidant defense mechanisms [18].

In a meta-analysis by Zhang et al., 13 RCTs with 786 participants found that prebiotics, probiotics, or synbiotics significantly improved depressive symptoms compared to placebo. The effects were seen in patients with mild to moderate depression, highlighting the potential of these interventions as adjunctive treatments. However, the authors emphasize the need for further clinical trials [19].

Many studies have identified a correlation between gut microbiota and iron deficiency anemia, with several reporting that probiotic supplementation may enhance iron absorption. Vonderheid et al. showed in a meta-analysis that *Lactiplantibacillus plantarum 299v* (*L. plantarum 299v*) improves iron absorption and hence assists in preventing iron-deficiency anemia [20]. Furthermore, Hoppe et al. observed that in patients with a greater requirement for iron supplementation, taking *L. plantarum 299v* together with a meal increases the bioavailability of iron [20]. Korcok et al. conducted research to investigate the additive effect of combining *L. plantarum 299v* with sucrosomal iron and vitamin C in the prevention and treatment of iron deficiency. The study involved two groups: one group received only vitamin C and iron supplementation, while the second group received additional supplementation with *L. plantarum*. Interestingly, it was observed that the second group, which additionally received *L. plantarum,* exhibited higher iron blood levels. This outcome was possibly attributed to the increased absorption of iron facilitated by the presence of *L. plantarum* [21].

By targeting these mechanisms, probiotics offer a comprehensive approach to managing depression and anemia, addressing both gut dysbiosis and the systemic factors that underlie these conditions. However, further research is required to properly understand which strains, dosages, and treatment periods are most beneficial for each illness.

The main objective of this secondary analysis was to evaluate the impact of probiotic supplementation on parameters related to RBCs in individuals suffering from depressive disorders. The secondary goal was to evaluate several potential pretreatment determinants of probiotic activity on RBCs, such as dietary habits, inflammatory or metabolic conditions, the severity and dimensions of psychiatric symptoms, and taken medications. The third goal was to evaluate probiotics’ effects on RBC parameters in addition to their effectiveness in treating depression.

We hypothesized that probiotic supplementation will positively influence RBC parameters, with these effects being modulated by the baseline inflammation or patients’ dietary intake. Furthermore, we proposed that improvements in RBC function will correlate with enhanced antidepressant outcomes, suggesting an integrative role of probiotics in both hematological health and mental well-being.

## 2. Materials and Methods

### 2.1. Design and Patients

The parent study was a parallel-group, prospective, randomized, double-blind, controlled, 60-day design [22]. This is a retrospective analysis of the study results regarding RBC-related parameters; the manuscript has been prepared according to the Consort statement [23].

Sample size was calculated for the primary outcome measure of the PRO-DEMET study and may be found elsewhere [22].

To be included in the research, the participants needed to have a diagnosis of depressive disorders based on the 11th International Classification of Diseases (ICD-11), such as dysthymia, recurrent depression, mixed depressive and anxiety disorder, or depressive episode. The Supplementary Material contains a detailed description of the remaining eligibility requirements and the study timeline.

An independent researcher randomized the patients and assigned them to either the probiotic (PRO) or placebo (PLC) treatments. Both the researchers and study participants were blinded. A computer-based random number generator was used to determine the randomization (https://www.randomizer.org, accessed on 10 December 2020; ClinicalTrials.gov identifier: NCT04756544).

### 2.2. Outcome Measures

Table 1 presents the outcome measures, with detailed explanations provided in the Supplementary Material (Table 1).

### 2.3. Questionnaires, Scales, Criteria

Basic data on sociodemographic and health-related information were gathered using a self-constructed survey questionnaire. Dietary habits were examined using the Food Frequency Questionnaire (FFQ-6) by Wądłowska. This questionnaire assesses the frequency of consumption of various food groups over a specified period [24]. The Montgomery–Åsberg Depression Rating Scale (MADRS) was used to determine depression symptom severity and dimensions. It evaluates ten key depressive symptoms, including apparent sadness, reported sadness, inner tension, reduced sleep, reduced appetite, concentration difficulties, lassitude, inability to feel, pessimistic thoughts, and suicidal thoughts [25]. The Depression, Anxiety, and Stress Scale (DASS) was used to assess the three negative emotional states of the study participants. This scale includes separate subscales for depression, anxiety, and stress, each measuring the severity of these states independently [26]. Quality of life was evaluated using the WHOQOL-BREF questionnaire. This instrument assesses the physical, psychological, social, and environmental domains of well-being [27].

### 2.4. Biological Material

Fasting venous blood was obtained by licensed nurses after overnight rest, in the morning, at the beginning (V1) and end (V2) of the intervention period. Laboratory parameters were measured in the Department of Laboratory Diagnostics, Central Teaching Hospital, and the Department of Biomedicine and Genetics, Medical University of Lodz, Poland.

### 2.5. Intervention

The probiotic group, known as PRO, consumed one capsule daily that contained 3 × 10^9^ CFU of *Lactobacillus helveticus Rosell^®^-52* and *Bifidobacterium longum Rosell^®^-175*, along with excipients (Sanprobi Stress^®^, Sanprobi Sp. z o. o., Sp. k., Szczecin, Poland; the probiotic powder was manufactured by Institute Rosell-Lallemand, located in Montreal, Canada). The placebo group (PLC) received the same capsule with only the excipients (manufacturer: Sanprobi Sp. z o. o., Sp. k., Szczecin, Poland).

For 60 days, the probiotic group (PRO) received a daily capsule containing a probiotic blend with 3 × 10^9^ colony-forming units (CFU) *of Lactobacillus helveticus Rosell^®^-52* and *Bifidobacterium longum Rosell^®^-175*, along with excipients (Sanprobi Stress^®^, Sanprobi Sp. z o. o., Sp. k., Szczecin, Poland; probiotic powder manufacturer—Institute Rosell-Lallemand, Montreal, Canada). The PLC group received one identical capsule with only the excipients (Sanprobi Sp. z o. o., Sp. k., Szczecin, Poland). The specific formulation of the probiotic was determined based on findings from our previous research [22].

### 2.6. Data Management

The data were cataloged in accordance with the Findability, Accessibility, Interoperability, and Reusability (FAIR) standards. Regarding the General Data Protection Regulation (EU) 2016/679 (GDPR), each study participant gave their informed consent before any data were processed for any purpose.

### 2.7. Ethics

The study was performed in compliance with the Declaration of Helsinki, and permission was obtained from the Medical University of Lodz’s Bioethics Committee (number RNN/109/20/KE dated 28 April 2020).

### 2.8. Statistical Analyses

Statistical analyses were conducted using Julia v1.10.4. Descriptive statistics, including means, standard deviations, medians, interquartile ranges, and confidence intervals, were calculated for continuous variables. For quantitative variables, the number of patients and percentages were provided. Listwise deletion was used to address missing data, which varied from 0% to 6.2% across most variables. The authors determined that this degree of missing data was unlikely to cause significant bias. Notably, no data were missing for any of the RBC parameters. The normality of distributions was assessed visually (Q-Q plots, histograms) and using the Kolmogorov–Smirnov test. Equality of variances was tested using the F test and, depending on its results, a respective (equal- or unequal-variance) *t*-test was used. Effect size was measured using Cohen’s d. Discrete variables were compared using the Χ^2^ test. Correlations were tested using Pearson correlation and an appropriate test for the inter-group coefficients. Linear regression models were tested to assess the effects of all major continuous clinical variables on the changes (post- minus pre-intervention values) in RBC parameters. Each model included the study group and one variable (with and without an interaction between the group and tested variable). Similarly, ANOVA was used to assess the effects of all major categorical clinical variables. Again, the ANOVA models included the study group and one variable (with and without an interaction between the group and tested variable). All statistical tests were conducted with a significance threshold set at *p* < 0.05 (two-sided). Given the multiple outcome measures in our study, we selected a single primary outcome and utilized point estimates with 95% confidence intervals and effect size metrics whenever feasible.

## 3. Results

**Figure 1:**
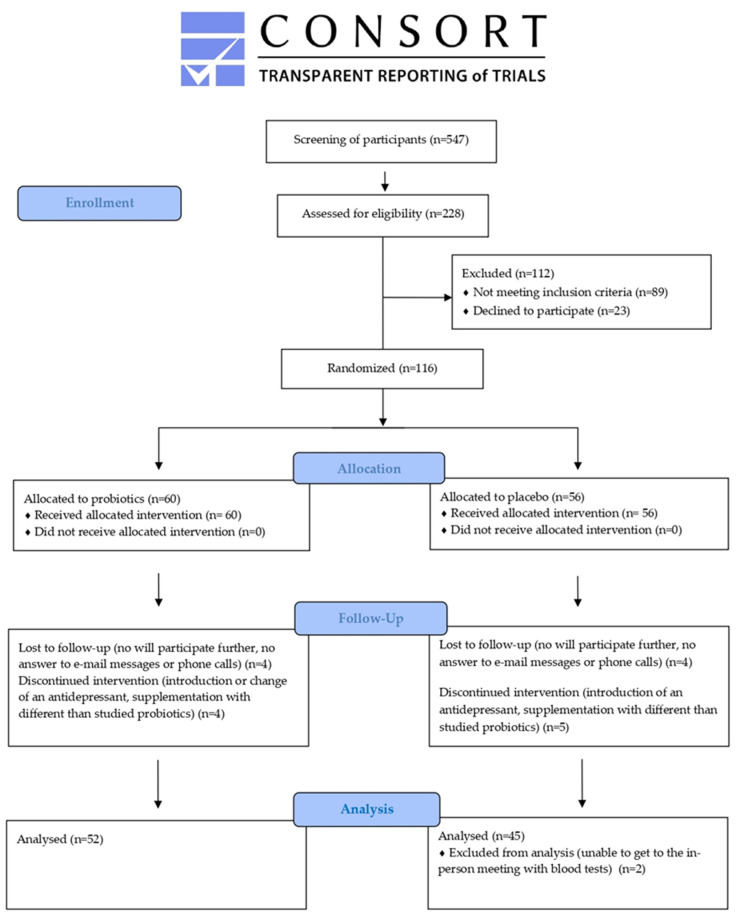
The study flow diagram. Abbreviations: PRO—probiotic; PLC—placebo; V2—the end of the intervention period.

Adult patients aged 18 and older were recruited for this study. A primary inclusion criterion was a confirmed diagnosis of depressive disorders, such as a depressive episode, recurrent depression, or a mixed depressive–anxiety disorder. Additional inclusion criteria included being at least 18 years old, having a Montgomery–Åsberg Depression Rating Scale (MADRS) score of 13 or higher, and having had no changes in antidepressant or antianxiety medications for at least three weeks prior to the study’s start. The exclusion criteria are detailed in the Supplementary Material. Figure 1 illustrates the study flow diagram.

### 3.1. Sample Characteristics

Sample characteristics were assessed by analyzing participants’ general characteristics, RBC parameters, dietary habits, psychometric measures, inflammatory markers, metabolic profiles, and other relevant factors (Table 2).

### 3.2. The Change in Red Blood Cell Parameters

Probiotic intake did not significantly alter the levels of red blood cell parameters, including RBC count, HCT, HGB, MCV, MCHC, and RDW, in comparison to the placebo (Table 3).

### 3.3. Possible Factors Influencing Probiotic Action

To identify variables for multivariate analysis, we conducted correlation analysis between changes in RBC-related parameters and baseline data (Table 4).

An analysis stratified by gender revealed no significant differences in any parameters between the PRO and PLC groups.

A set of linear regression models were constructed for the following outcomes: ΔRBC, ΔHGB, ΔHCT, ΔMCV, ΔMCH, ΔMCHC, and ΔRDW. Each model included the study group (PRO or PLC) and one independent variable. Models with an interaction between the study group and independent variable and without that interaction were tested. After applying Bonferroni correction for multiple comparisons, none of the regression models were statistically significant. In a similar manner, we ran a two-way ANOVA analysis. In the ANOVA models, the dependent variables were ΔRBC, ΔHGB, ΔHCT, ΔMCV, ΔMCH, ΔMCHC, and ΔRDW. Each ANOVA model included the study group and one categorical variable (with and without an interaction between the group and tested variable). Again, none of the ANOVA models were statistically significant (Bonferroni correction for multiple comparisons was also applied).

### 3.4. Correlation Analysis Between RBC-Related Changes and the Changes in Psychometric Parameters

Finally, correlation analysis on the magnitude of changes between RBC-related and psychometric parameter values [28] was conducted (Table 5). The table revealed some possible patterns and moderate correlations between changes in hematological parameters and psychological assessment scores, different in PRO compared to the PLC group.

In the PLC group, increases in RBC counts and HCT levels were associated with a deterioration in self-assessed depressive and anxiety symptoms. Furthermore, this group also exhibited a positive correlation between MCH and MCHC changes and differences in MADRS scores.

## 4. Discussion

Despite the proposed benefits of probiotics in modulating RBC parameters as a potential mechanism for alleviating depression, our findings did not support this relationship. This study provided limited evidence of significant changes in RBC parameters following probiotic supplementation, thereby leading to a rejection of the initial hypothesis. This suggests that while probiotics may have therapeutic potential in depression, their impact on RBC markers appears minimal under the conditions studied.

### 4.1. The Interplay of Demographic and Clinical Diversity in Probiotic Outcomes

One possible explanation for the lack of meaningful changes is the diversity of patients’ reactions to probiotic intervention. Individual differences in baseline gut microbiota composition may result in varying responses to probiotic administration. Studies have indicated that the gut microbiome profiles of patients with depression differ from those of healthy individuals in several cases. Depressed individuals frequently exhibit microbiota dysbiosis, which is characterized by decreased beneficial bacteria and an increase in harmful ones. This imbalance may enhance intestinal permeability, allowing bacterial endotoxins to enter the bloodstream, inducing systemic inflammation [13,29,30,31,32]. Chronic inflammation, in turn, is a known contributor to conditions like anemia of chronic disease, which affects RBC production and lifespan [33]. These systemic implications of dysbiosis highlight the difficulty of alleviating its detrimental effects only through therapies like probiotics.

Furthermore, the diversity among patients in terms of age, gender, medication regimens, dietary supplements, and comorbidities complicates the ability to observe meaningful changes in RBC parameters through probiotic interventions. Age-related physiological changes and hormonal differences between genders can significantly impact gut microbiota composition, immune responses, and overall health outcomes [34,35,36,37], contributing to the heterogeneous nature of study populations and potentially influencing the response to probiotics. Additionally, depressed individuals frequently take drugs that can exert substantial direct effects on physiological processes, including blood composition, potentially overshadowing any subtle effects induced by probiotics [38].

### 4.2. The Role of Dietary Habits and Nutrient Absorption in Treatment Efficacy

When assessing the effect of probiotic supplementation on RBC parameters in patients with depressive disorders, it is essential to account for factors such as dietary deficiencies, impaired nutrient absorption, and coexisting conditions like gastrointestinal diseases or chronic inflammation. These factors can interfere with RBC synthesis and nutrient uptake, potentially limiting the efficacy of probiotics [39].

Probiotic management alone may not be adequate to significantly enhance RBC parameters if patients’ diets are insufficient in crucial nutrients required for erythropoiesis [40]. In our study, the dietary habits of the PRO and PLC groups were found to be largely comparable across all examined categories, which indicates that diet did not significantly interfere with the study’s outcomes.

### 4.3. The Impact of Health Comorbidities on Probiotic Treatment Outcomes

Moreover, individuals with depression commonly exhibit diverse comorbidities such as diabetes, cardiovascular diseases, and autoimmune disorders [41,42,43,44,45,46]. These conditions have their own physiological implications, including implications for inflammation and metabolism, which might influence RBC parameters and disturb the assessment of probiotic efficacy.

Regarding chronic inflammation, in our study, the comparison of inflammation parameters between the PRO and PLC groups revealed that, apart from lymphocyte counts (LYMs), there were no significant differences in inflammation markers (CRP, WBC, NEU, MON, PLT, SII, and TNF-α levels) at the beginning of the trial. Moreover, inflammatory markers were not shown to influence intervention outcomes in our secondary analyses. This suggests that inflammation likely did not affect the changes in RBC parameters.

### 4.4. The Importance of Probiotic Strain, Duration, and Dosage in Therapeutic Outcomes

Critical factors to consider are the probiotic intervention’s strains, length, and dosage [47]. The intervention duration may have been insufficient, as erythropoiesis, the complex process of the production of red blood cells, may require prolonged intervention to manifest measurable outcomes [48]. Therefore, extending the duration of probiotic supplementation in future research could provide a clearer picture of its impact on RBC parameters.

Furthermore, the dose and specific strains of probiotics used in the study may have been inappropriate. Probiotics have a wide range of strain-specific effects, and different strains may have an independent ability to influence physiological systems in addition to their core gastrointestinal properties [49,50]. However, if the selected probiotic strain lacks the necessary immunomodulatory properties or does not adequately interact with the gut-associated immune system, it may not produce the desired effect on hematological parameters. There is substantial evidence indicating that certain probiotic strains, including *Lactobacillus acidophilus* and *L. plantarum* 299v, enhance iron absorption and positively influence anemia management. While these findings are promising, further research is imperative to expand our understanding of additional strains and their mechanisms. Comparative studies on varied dosages and strain combinations are essential to elucidate the most effective probiotic interventions for optimizing RBC parameters and treating anemia more comprehensively [15,20].

### 4.5. The Associations Between the Changes in RBC-Related Parameters and the Changes in Psychological Symptoms

It is important to discuss the correlations in the PRO and PLC groups regarding the changes in RBC parameters along with the differences in psychological symptoms following the intervention.

In the PLC group, increased RBC count and HCT level were linked to worsened depressive symptoms. This implies a possible association between elevated RBC parameters and depressive symptoms in the absence of probiotic intervention. Moderate positive correlations were also noted between changes in MCH and MCHC with changes in depressive symptoms, as measured by MADRS scores. These findings suggest a meaningful relationship between these hematological parameters and mood changes when probiotics are not administered, indicating that these parameters may be naturally associated with depressive symptom fluctuations.

This may be due to a combination of physiological and behavioral factors commonly seen in depression, such as chronic stress and inflammation. Chronic stress activates the hypothalamic–pituitary–adrenal (HPA) axis, raising cortisol levels and potentially stimulating erythropoietin (EPO) production in the kidneys, which drives RBC production. Furthermore, chronic inflammation in depression activates pro-inflammatory cytokines like IL-6 and TNF-α, which not only contribute to the stress response but may also promote erythropoiesis by affecting the bone marrow environment [51,52].

The moderate positive correlations noted between changes in MCH and MCHC with depressive symptoms may also be explained by several mechanisms. Chronic inflammation, commonly seen in depression, can disrupt RBC production and alter MCH and MCHC levels through the action of inflammatory cytokines [53,54]. Additionally, nutritional deficiencies often seen in depression, such as those in vitamin B12 and folate, may alter MCH and MCHC levels [55,56]. Moreover, stress dysregulation, including elevated cortisol levels, may influence MCH and MCHC values by affecting erythropoiesis and the balance of hemoglobin within RBCs. This dysregulation is a common feature of the HPA axis in depression, potentially leading to further imbalances in these RBC parameters in the PLC group [57]. Together, these factors may drive the associations observed between RBC count, HCT, MCH, and MCHC in the PLC group, highlighting the complex interplay that influences both hematological parameters and depressive symptoms. In contrast, probiotic supplementation may help break this cycle, potentially stabilizing these parameters and their relationship with mood.

In the PRO group, these associations between RBC parameters and depressive symptoms were not observed. This finding implies that probiotic supplementation may help stabilize RBC, HCT, MCH, and MCHC levels, potentially preventing fluctuations in these parameters that correlate with depressive symptoms in the PLC group.

The lack of significant correlations in the PRO group compared to the PLC group suggests that probiotics might prevent adverse alterations in RBC parameters through several potential mechanisms.

Probiotics may reduce systemic inflammation by modulating gut microbiota, leading to decreases in pro-inflammatory cytokines. They may also stimulate the production of anti-inflammatory cytokines, such as interleukin-10 (IL-10) and transforming growth factor-beta (TGF-β), while simultaneously reducing levels of pro-inflammatory markers like TNF-α, IL-6, and CRP [58,59,60,61]. Since pro-inflammatory cytokines can drive excessive erythropoiesis, their reduction through probiotic supplementation could help normalize RBC and HCT levels. This reduction would break the link observed in the PLC group between elevated RBC and HCT levels and depressive symptoms [52,62]. By alleviating inflammation, probiotics may not only normalize elevated RBC and HCT levels but also help stabilize other RBC parameters, such as MCH and MCHC, which have also shown correlations with mood changes. Stabilizing these hematological parameters may help prevent the pattern where physiological imbalances contribute to depressive symptoms. By reducing fluctuations in RBC and HCT levels, probiotics could provide a more consistent physiological environment, potentially leading to a reduction in depressive symptoms.

Probiotics can support RBC health and function by reducing oxidative stress. They achieve this by enhancing endogenous antioxidant enzymes that neutralize reactive oxygen species and generating antioxidants like glutathione, folate, and short-chain fatty acids, reducing oxidative damage [63]. By reducing oxidative stress, probiotics help to stabilize RBC, HCT, MCH, and MCHC levels.

Furthermore, probiotics may influence RBC parameters through their effects on the gut–brain axis and the HPA axis. By modulating the gut microbiota and promoting beneficial bacterial species that produce signaling molecules such as SCFAs, probiotics can help regulate the HPA axis, leading to a reduction in cortisol and erythropoietin (EPO) production. This reduction stabilizes RBC and HCT levels [57,64]. Additionally, probiotics may influence neurotransmitter synthesis [65] and enhance blood–brain barrier integrity [66], potentially reducing central nervous system inflammation and stress, which further contributes to stable cortisol levels and reduced erythropoiesis.

Additionally, by improving gut health, probiotics might enhance the absorption of essential nutrients like vitamin B12 and folate, which are critical for RBC production and the maintenance of MCH and MCHC levels [67]. This improved nutrient absorption may contribute to appropriate RBC, HCT, MCH and MCHC levels, supporting both hematological health and mood stabilization.

Probiotics may possess the potential to influence both hematological parameters and depressive symptoms by potentially reducing inflammation, oxidative stress, and cortisol levels, while also enhancing nutrient absorption and modulating the gut–brain axis. However, the exact pathways through which probiotics exert these effects remain unclear, highlighting the need for further research to elucidate these mechanisms and validate the therapeutic potential of probiotics.

### 4.6. Measurement Sensitivity and Limitations

This study has several notable limitations that warrant careful consideration when interpreting the results. The instruments used to measure changes in hematological parameters might have inherent limitations regarding their sensitivity and specificity. Hematological parameters display daily variability influenced by factors like hydration status, recent diet, physical activity, and the timing of blood sample collection. Failure to account for these fluctuations can disturb data. However, in our patients, the timing of blood sample collection was consistent across all participants, thereby ensuring that this factor did not introduce variability or disturb our data. Additionally, the precision and accuracy of measurement techniques are critical; even minor measurement errors or equipment calibration issues can introduce variability that might obscure genuine correlations.

A notable limitation of our study is the lack of assessment of the input status of essential nutrients, specifically iron, vitamin B12, and vitamin B9, which are critical determinants of RBC parameters [55]. The absence of data on these micronutrient levels may affect the accuracy and interpretability of our hematological findings, as variations or deficiencies in these nutrients can significantly impact RBC counts and function.

### 4.7. The Need for Expanded Probiotic Research and Thorough Evaluation

In future studies, increasing the sample size and ensuring a more homogenous population will be crucial for enhancing statistical power to detect subtle correlations effectively. This approach will also help minimize the influence of random variability, improve the generalizability of findings, provide more precise estimates of relationships between variables, facilitate subgroup analyses, and ensure greater reliability and reproducibility of research outcomes.

Clinicians should evaluate patients’ dietary habits, underlying gastrointestinal health, and nutritional status before introducing probiotic therapies to maximize therapeutic success in subsequent trials. Longitudinal studies that examine the combined effects of probiotics, dietary adjustments, and nutrient supplementation have the potential to unveil the most efficacious strategies for enhancing RBC-related parameters in individuals with depressive disorder.

## 5. Conclusions

In summary, the lack of evident alterations in RBC-related parameters after probiotic administration in individuals with depressive disorder highlights the challenges associated with comprehending the interplay of gut-related interventions, depression, and physiological health markers. However, the correlations identified in our study, particularly those involving RBC count and HCT, indicate several promising avenues for future investigation. These findings suggest that further research could elucidate the intricate relationships between these RBC parameters, probiotics, and depressive symptoms, potentially leading to more effective therapeutic strategies.

## Figures and Tables

**Table 1:** The trial outcome measures.

Outcome Measures	
Primary	ΔHGB
Secondary	ΔRBC, ΔHCT, ΔMCV, ΔMCH, ΔMCHC, ΔRDW
Tertiary	Baseline: weight, BMI, WC, WWI, WHtR, BP, fGlc, HDL-c, non-HDL-c, TGs, ALT, AST, TG/HDL-c, ALT/AST, HSI, CLGI presence, CRP, NEUs, LYMs, MONs, PLTs, NLR, MLR, PLR, SII, I-FABP, TNF-α, dietary habits, physical activity level, antidepressant treatment, MADRS, DASS, QoLOthers: %ΔMADRS, MCID MADRS, CMC MADRS, response MADRS, remission MADRS, %ΔDASS, MCID DASS

Abbreviations: ALT—alanine aminotransferase; AO—abdominal obesity; AST—aspartate aminotransferase; BP—blood pressure; CLGI—chronic low-grade inflammation; CMC—clinically meaningful change; CRP—C-reactive protein; DASS—Depression, Anxiety, and Stress Scale; fGlc—fasting glucose; HDL-c—high-density lipoprotein cholesterol; HSI—Hepatic Steatosis Index; I-FABP—intestinal fatty acid-binding protein; LYMs—lymphocytes; MADRS—Montgomery–Åsberg Depression Rating Scale; MCID—minimum clinically important difference; MetS—metabolic syndrome; MLR—MON/LYM; MONs—monocytes; NEUs—neutrophils; NLR—NEU/LYM; PLTs—platelets; QoL—quality of life; SII—Systemic Inflammatory Index; TGs—triglycerides; WC—waist circumference; WHtR—waist-to-height ratio; WWI—weight-adjusted waist index; Δ—difference between the end (V2) and the beginning (V1) of the intervention period; %Δ—percentage Δ.

**Table 2:** Characteristics of the study participants at the beginning of the trial.

Characteristics	Total (*n* = 97)		PRO Group (*n* = 52)		PLC Group (*n* = 45)		Missing Data	*p*
Sex (F:M)	83:14		44:8		39:6		0	0.593
Age (y) (mean ± SD)	34.35 ± 13.38		34.89 ± 14.54		33.72 ± 12.03		0	0.667
Diagnosis (6A70:6A71:6A73)	9:27:62		7:17:29		2:10:33		0	0.301
Psychotropic medications (%)	69.07		69.23		68.89		0	0.541
Antidepressants (%)	69.07		69.23		68.89		0	0.541
Antipsychotics (%)	4.12		5.77		2.22		0	0.317
Comorbidities (%)	51.55		55.77		46.67		0	0.258
Pharmacological treatment other than psychotropics (%)	35.05%		40.38%		28.89%		0	0.170
Smoking cigarettes (%)	14.43%		11.54%		17.78%		0	0.593
Dietary supplements (%)	52.58%		59.62%		44.44%		0	0.123
Physical activity (MET-min/week) (mean ± SD)	1975.38 ± 1434.74		2055.72 ± 1577.96		1886.11 ± 1296.64		60.82	0.721
**Red blood cell parameters**								
	**Total**		**PRO**		**PLC**		**Missing data**	***p***
	Mean	SD	Mean	SD	Mean	SD		
RBCs (×10^6^/µL)	4.58	0.38	4.62	0.35	4.54	0.41	0	0.656
HCT (%)	39.84	3.06	39.91	3.14	39.76	2.99	0	0.781
HGB (g/dl)	13.44	1.26	13.45	1.34	13.43	1.17	0	0.491
MCV (fl)	87.15	4.54	86.56	4.75	87.84	4.23	0	0.443
MCHC (g/dl)	33.69	1.14	33.64	1.19	33.75	1.09	0	0.949
RDW (%)	13.21	1.27	13.25	1.33	13.17	1.22	0	0.933
**Dietary habits** Food frequency intake assessed on a scale 1–6: 1—never or almost never; 2—once a month; 3—several times a month; 4—several times a week; 5—every day; 6—several times a day.								
	**Total**		**PRO**		**PLC**		**Missing data (%)**	***p***
	Mean	SD	Mean	SD	Mean	SD		
Sweets and snacks	2.63	0.68	2.54	0.67	2.75	0.70	2.06	0.133
Dairy and eggs	3.08	0.73	2.98	0.67	3.20	0.79	2.06	0.152
Cereal products	3.10	0.57	3.05	0.53	3.16	0.61	2.06	0.324
Oils	2.62	0.59	2.60	0.60	2.64	0.59	2.06	0.784
Fruits	2.78	0.52	2.72	0.49	2.85	0.56	2.06	0.246
Vegetables and seeds	3.36	0.59	3.27	0.50	3.47	0.66	2.06	0.093
Meat (including fish)	2.28	0.67	2.30	0.64	2.25	0.72	2.06	0.714
Drinks (excluding water)	2.05	0.56	2.00	0.52	2.10	0.60	2.06	0.375
Processed food products	2.40	0.45	2.34	0.45	2.48	0.45	2.06	0.136
**Psychometric parameters**								
	**Total**		**PRO**		**PLC**		**Missing data (%)**	***p***
	Mean	SD	Mean	SD	Mean	SD		
MADRS score	19.99	5.07	20.50	5.36	19.40	4.71	0	0.289
Sadness	4.38	1.73	4.38	1.63	4.40	1.85	6.19	0.956
Neurovegetative symptoms	5.36	2.21	5.88	2.27	4.79	1.81	6.19	0.014
Detachment	7.10	2.19	7.04	2.32	7.16	2.06	6.19	0.794
Negative thoughts	3.14	1.38	3.08	1.43	3.21	1.34	6.19	0.666
DASS score	63.64	21.66	62.55	22.52	64.91	20.81	2.06	0.599
Depression	20.92	9.56	20.04	10.39	21.93	8.51	2.06	0.339
Anxiety	17.26	8.50	17.37	9.06	17.14	7.90	2.06	0.893
Stress	25.46	8.45	25.14	8.20	25.84	8.82	2.06	0.688
QoL score	73.40	12.39	74.88	12.56	71.68	12.12	2.06	0.211
Physical	18.85	4.01	18.73	4.03	19.00	4.03	2.06	0.742
Psychological	15.41	3.55	15.73	3.64	15.05	3.44	2.06	0.354
Social	8.58	2.37	8.78	2.34	8.34	2.40	2.06	0.366
Environment	25.11	4.71	26.08	4.77	23.98	4.44	2.06	0.030
**Inflammation parameters**								
	**Total**		**PRO**		**PLC**		**Missing data**	***p***
	Mean	SD	Mean	SD	Mean	SD		
CRP (mg/l)	2.0	2.03	2.01	1.95	2.0	2.13	0	0.981
WBCs (×10^3^/µL)	6.08	1.4	5.97	1.36	6.21	1.46	0	0.410
NEUs (×10^3^/µL)	3.36	1.03	3.34	1.09	3.39	0.97	0	0.792
LYMs (×10^3^/µL)	1.99	0.52	1.89	0.46	2.1	0.56	0	0.039
MONs (×10^3^/µL)	0.51	0.15	0.52	0.15	0.5	0.14	0	0.484
PLTs (×10^3^/µL)	278.27	53.19	277.6	54.68	279.04	52.02		0.894
SII	495.06	217.45	515.51	248.23	471.43	175.15		0.310
TNF-α (pg/mL)	28.32	112.33	29.94	123.7	26.49	99.23	1.03	0.881
**Metabolic parameters**								
	**Total**		**PRO**		**PLC**		**Missing data (%)**	***p***
	Mean	SD	Mean	SD	Mean	SD		
Weight (kg)	70.45	15.38	69.42	15.09	71.64	15.79	0	0.481
BMI (kg/m^2^)	24.79	4.61	24.3	4.07	25.37	5.14	0	0.256
WC (cm)	85.08	13.4	84.34	12.4	85.94	14.56	0	0.558
WWI (cm/√kg)	10.16	0.82	10.15	0.76	10.17	0.88	0	0.926
WHtR (cm/cm)	0.5	0.08	0.5	0.07	0.51	0.09	0	0.442
sBP (mmHg)	121.47	14.08	122.06	13.88	120.8	14.45	0	0.663
dBP (mmHg)	82.13	8.76	82.6	8.96	81.6	8.6	0	0.579
fGlc (mmol/l)	5.17	0.53	5.17	0.51	5.17	0.56	0	0.959
HDL-c (mmol/l)	1.64	0.35	1.71	0.37	1.57	0.31	0	0.042
non-HDL-c	3.71	1.02	3.75	1.03	3.67	1.0	0	0.716
TGs (mmol/l)	1.16	0.64	1.13	0.67	1.19	0.61	0	0.644
ALT	21.4	15.09	21.75	14.12	21.0	16.29	0	0.810
ALT/AST	0.84	0.34	0.85	0.39	0.82	0.29	0	0.644
HSI	33.19	6.2	32.85	6.17	33.58	6.28	0	0.568
FSI	−2.46	1.45	−2.52	1.45	−2.38	1.46	0	0.639
APRI	0.26	0.14	0.26	0.09	0.27	0.18	0	0.981
FIB-4	0.71	0.36	0.73	0.38	0.69	0.33	0	0.577
**Others**								
	**Total**		**PRO**		**PLC**		**Missing data (%)**	***p***
	Mean	SD	Mean	SD	Mean	SD		
I-FABP (ng/mL)	1966.16	1252.2	2039.23	949.71	1879.8	1542.84	1.03	0.552

Abbreviations: ALT—alanine aminotransferase; AST—aspartate aminotransferase; APRI—aspartate aminotransferase-to-platelet ratio index; BMI—body mass index; bSCFAs—branched short-chain fatty acids; CRP—C-reactive protein; DASS—Depression, Anxiety, and Stress Scale; dBP—diastolic blood pressure; F—female; fGlc—fasting glucose; FIB-4—fibrosis index based on four factors; HCT—hematocrit; HDL-c—HDL cholesterol; HGB—hemoglobin; HSI—Hepatic stenosis index; I-FABP—intestinal fatty acid-binding protein; LYMs—lymphocytes; M—male; MADRS—Montgomery–Åsberg Depression Rating Scale; MCH—mean hemoglobin concentration; MCHC—mean corpuscular hemoglobin concentration; MCV—mean corpuscular volume; MONs—monocytes; NEUs—neutrophils; Non-HDL—non-high-density lipoprotein cholesterol; PLC—placebo; PLTs—platelets; PRO—probiotic; QoL—quality of life; RBCs—red blood cells; RDW—RBC distribution width; sBP—systolic blood pressure; SD—standard deviation; SII—systemic inflammatory index; TGs—triglycerides; TNF-α—tumor necrosis factor alpha; WBCs—white blood cells; WC—waist circumference; WHtR—waist-to-height ratio; WWI—weight-adjusted waist index; y—years; 6A70—depressive episode; 6A71—recurrent depression; 6A73—mixed depressive and anxiety disorder.

**Table 3:** The influence of probiotic intervention on RBC-related parameters. N = 97.

Parameter	V1 PRO (Mean ± SD)	V2 PRO (Mean ± SD)	∆PRO (Mean ± SD)	V1 PLC (Mean ± SD)	V2 PLC (Mean ± SD)	∆PLC (Mean ± SD)	*p*	Difference in Mean (PRO—PLC) for Δ [95%CI]	Cohen’s d [95% CI]
HGB	13.45 ± 1.34	13.30 ± 1.41	−0.15 ± 0.54	13.43 ± 1.34	13.42 ± 1.19	−0.01 ± 0.71	0.268	−0.14 [−0.39, 0.11]	0.07 [−0.22, 0.35]
RBCs	4.62 ± 0.35	4.58 ± 0.35	−0.04 ± 0.18	4.54 ± 0,35	4.51 ± 0.48	−0.03 ± 0.24	0.714	−0.02 −0.1, 0.07]	0.09 [−0.20, 0.37]
HCT	39.91 ± 3.14	39.62 ± 3.25	−0.29 ± 1.61	39.76 ± 3.00	39.64 ± 3.42	−0.12 ± 2.23	0.665	−0.17 [−0.95, 0.61]	0.07 [−0.22, 0.35]
MCV	86.56 ± 4.76	86.69 ± 4.84	0.14 ± 1.28	87.84 ± 4.23	88.18 ± 4.49	0.33 ± 2.02	0.560	−0.20 [−0.87, 0.48]	−0.05 [−0.33, 0.23]
MCH	29.16 ± 2.32	29.05 ± 2.38	−0.11 ± 0.57	29.66 ± 1.94	29.96 ± 3.67	0.30 ± 2.15	0.190	−0.41 [−1.02, 0.21]	−0.03 [−0.31, 0.25]
MCHC	33.64 ± 1.19	33.50 ± 1.20	−0.14 ± 0.57	33.75 ± 1.09	33.93 ± 2.49	0.18 ± 1.72	0.210	−0.320 [−0.82, 0.18]	−0.004 [−0.29, 0.28]
RDW	13.25 ± 1.33	13.29 ± 1.42	0.04 ± 0.58	13.17 ± 1.22	13.37 ± 1.61	0.20 ± 0.76	0.253	−0.16 [−0.43, 0.11]	−0.08 [−0.36, 0.20]

Abbreviations: CI—confidence interval; HCT—hematocrit; HGB—hemoglobin; MCH—mean hemoglobin concentration; MCHC—mean corpuscular hemoglobin concentration; MCV—mean corpuscular volume; PLC—placebo; PRO—probiotic; RBCs—red blood cells; RDW—RBC distribution width; SD—standard deviation; ∆—change between the end and the start of the intervention.

**Table 4:** A heatmap illustrating correlations between changes (Δ) in inflammatory parameters and selected pre-treatment data. * *p* < 0.05.

         r≥0.400.30 to 0.390.20 to 0.290.10 to 0.19−0.09 to 0.09−0.10 to −0.19−0.20 to −0.29−0.30 to −0.39≤−0.40

PRO										
	CRP	TNF-α	fGlc	sBP	WC	TGs	HDL	Non-HDL	I-FABP	bSCFAs
**Δ HGB**	−0.14	−0.06	0.15	0.08	0.16	−0.09	0.02	−0.11	−0.23	−0.09
**Δ RBCs**	−0.01	−0.09	0.14	0.07	0.14	−0.11	−0.10	−0.12	−0.10	0.00
**Δ HCT**	−0.13	−0.04	0.11	0.04	0.14	−0.06	−0.11	−0.14	−0.15	0.00
**Δ MCV**	−0.21	0.14	−07	−0.11	−0.03	0.08	0.05	−0.04	−0.15	0.02
**Δ MCH**	−0.07	−0.02	0.03	−0.07	0.07	0.07	0.14	−0.04	−0.32 *	−0.22
**Δ MCHC**	0.05	−0.10	0.12	0.13	0.07	−0.04	0.28 *	0.08	−0.24	−0.24
**Δ RDW**	−0.07	0.01	0.11	−0.02	−0.16	−0.01	−0.40 *	−0.19	0.27	0.14
**PLC**										
	**CRP**	**TNF-α**	**fGlc**	**sBP**	**WC**	**TGs**	**HDL**	**Non-HDL**	**I-FABP**	**bSCFAs**
**Δ HGB**	0.25	0.04	−0.09	0.02	0.05	0.01	0.05	0.06	−0.10	0.00
**Δ RBCs**	0.36 *	−0.03	−0.01	0.15	0.19	0.22	−0.06	0.34 *	−0.13	0.04
**Δ HCT**	0.36 *	0.00	−0.01	0.10	0.25	0.14	−0.07	0.32 *	−0.10	0.06
**Δ MCV**	0.04	0.11	0.01	−0.11	0.16	−0.11	−0.04	−0.04	0.05	−0.06
**Δ MCH**	−0.15	0.05	0.44	0.15	0.20	0.10	0.23	0.01	0.02	−0.07
**Δ MCHC**	−0.18	0.04	−0.13	−0.18	−0.28	−0.21	0.20	−0.38 *	0.01	−0.09
**Δ RDW**	0.01	−0.21	−0.17	−0.27	−0.05	−0.13	0.15	−0.20	0.10	0.01

Abbreviations: bSCFAs—branched short-chain fatty acids; CRP—C-reactive protein; fGlc—fasting glucose; HCT—hematocrit; HDL-c—high-density lipoprotein cholesterol; HGB—hemoglobin; I-FABP—intestinal fatty acid-binding protein; MCH—mean hemoglobin concentration; MCHC—mean corpuscular hemoglobin concentration; MCV—mean corpuscular volume; Non-HDL—non-high-density lipoprotein cholesterol; RBCs—red blood cells; RDW—RBC distribution width; sBP—systolic blood pressure; TGs—triglycerides; TNF-α—tumor necrosis factor alpha; WC—waist circumference.

**Table 5:** Correlation analysis of the percentage change in RBC-related markers and in psychometric scale scores. * *p* < 0.05; red—greater improvement in a psychometric scale is a negative predictor of a decrease in RBC-related parameter levels; green—greater improvement in a psychometric scale is a positive predictor of a decrease in RBC-related parameter levels.

         r≥0.400.30 to 0.390.20 to 0.290.10 to 0.19−0.09 to 0.09−0.10 to −0.19−0.20 to −0.29−0.30 to −0.39≤−0.40

	PRO				
	% ∆ MADRS	% ∆ DASS	% ∆ D-DASS	% ∆ A-DASS	% ∆ S-DASS
**Δ HGB**	0.26	0.08	0.08	0.13	0.02
**Δ RBCs**	0.26	0.08	0.09	0.11	0.03
**Δ HCT**	0.29*	0.09	0.10	0.14	0.05
**Δ MCV**	0.15	0.02	0.01	0.03	0.06
**Δ MCH**	0.13	0.03	0.01	0.07	0.02
**Δ MCHC**	−0.05	−0.01	−0.02	0.00	−0.07
**Δ RDW**	−0.08	−0.15	−0.01	−0.16	−0.25
	**PLC**				
	**% ∆ MADRS**	**% ∆ DASS**	**% ∆ D-DASS**	**% ∆ A-DASS**	**% ∆ S-DASS**
**Δ HGB**	0.30	0.18	0.23	0.17	0.08
**Δ RBCs**	−0.03	0.31	0.36 *	0.33 *	0.16
**Δ HCT**	0.02	0.33 *	0.36 *	0.32	0.20
**Δ MCV**	0.13	00.02	−0.01	−0.09	0.12
**Δ MCH**	0.34 *	−0.17	−0.17	−0.23	−0.08
**Δ MCHC**	0.35 *	−0.20	−0.19	−0.23	−0.14
**Δ RDW**	0.16	0.08	0.03	0.01	0.17

Abbreviations: A-DASS—Anxiety-DASS; DASS—Depression, Anxiety, and Stress Scale; D-DASS—Depression-DASS; HCT—hematocrit; HGB—hemoglobin; PLC—placebo; PRO—probiotic; MADRS—Montgomery–Åsberg Depression Rating Scale; MCH—mean hemoglobin concentration; MCHC—mean corpuscular hemoglobin concentration; MCV—mean corpuscular volume; RBCs—red blood cells; RDW—RBC distribution width; S-DASS—Stress-DASS; Δ—change between the end and the beginning of the intervention; %Δ—percentage Δ.

## Data Availability

The data that supports the findings of this study are available from the corresponding author, O.G.-K., upon reasonable request.

## Supplementary Materials

The following supporting information can be downloaded at https://www.mdpi.com/article/10.3390/jcm14010265/s1: The supplementary material for the manuscript has been included in the attached files [68,69,70,71,72,73,74,75,76,77,78,79,80,81,82,83,84,85,86,87,88,89,90,91,92,93,94,95].
