# Supplementary material for: Limited Changes in Red Blood Cell Parameters After Probiotic Supplementation in Depressive Individuals: Insights from a Secondary Analysis of the PRO-DEMET Randomized Controlled Trial
Source: J Clin Med. 2025 Jan 5;14(1):265. doi: 10.3390/jcm14010265 (PMC11721667; doi:10.3390/jcm14010265)
Supplement: Supplementary file 1 [file jcm-14-00265-s001.zip › jcm-3390487-supplementary.pdf]

1. The recruitment, randomization, and intervention

Adult patients aged 18 and older with depressive disorders, diagnosed according to the 11th International Classification of Diseases [68], were recruited for this study. Recruitment took place at psychiatric outpatient clinics in central Poland, through social media advertisements, and via a snowball sampling method. Participants were randomly assigned into two groups (1:1 ratio) using computer-generated blocked lists, stratified based on the presence of Metabolic Syndrome (MetS). This randomization process was conducted with the aid of a computer-based random number generator (https://www.randomizer.org/, accessed on December 10th, 2020). The probiotic group (PRO) for 60 days received a daily capsule containing a probiotic blend with 3×10^9 colony-forming units (CFU) of Lactobacillus helveticus Rosell®-52 and Bifidobacterium longum Rosell®-175, along with excipients (Sanprobi Stress®, Sanprobi Sp. z o. o., Sp. k., Szczecin, Poland; probiotic powder manufacturer—Institute Rosell-Lallemand, Montreal, Canada). The PLC group received one identical capsule with only the excipients (Sanprobi Sp. z o. o., Sp. k., Szczecin, Poland). The specific formulation of the probiotic was determined based on findings from our previous research [69].

2. Elligibility criteria

One of the main criteria for inclusion in the study was a confirmed diagnosis of depressive disorders, such as a depressive episode, recurrent depression, or mixed depressive and anxiety disorder. Additional criteria for inclusion included being over 18 years old, having a Montgomery-Asberg Depression Rating Scale (MADRS) score of 13 or higher, and no changes in antidepressant or antianxiety medications for at least three weeks before the start of the study. The exclusion criteria were as follows:

a. pregnancy,
b. an infection and/or vaccination and/or treatment with antibiotics, supplementation with probiotics or prebiotics, being diagnosed with or having new symptoms of autoimmune, serious immunocompromised, inflammatory bowel diseases, cancer, IgE-dependent allergy, a significant change in a dietary pattern, a significant change in dietary supplementation, a significant change in daily physical activity or an extreme sport activity, a significant change in a smoking pattern, a significant change in the treatment schema with proton-pump inhibitors, metformin, laxatives, systemic steroids, nonsteroidal anti-inflammatory drugs, antipsychotics, or any other medications influencing the microbiota according to present knowledge in the previous four weeks,
c. current decompensated serious somatic disease, psychiatric comorbidities (except for a specific personality disorder, an additional specific anxiety disorder, and caffeine or nicotine addiction),
d. a major neurological disorder or any medical disability that may interfere with a subject's ability to complete the study procedures,
e. high risk of suicide,
f. current or recent participation in another research study involving an intervention that may alter outcomes that are relevant for this study.

The criteria were developed considering factors known to impact depression, inflammation, metabolic health, and the state of the microbiota.

3. Study timeline

- V0: Eligibility criteria, the study questionnaire (SQ) and the MADRS completion, informed consent, full psychiatric examination.
- V1 (no longer that five days after V0), "randomization visit": Depression, Anxiety and Stress Scale (DASS), The World Health Organization quality of life-BREF (WHOQOL-BREF) completion, Food Frequency Questionnaire-6 (FFQ-6), blood pressure (BP), body mass index (BMI), waist circumference (WC) measurements, blood collection.

- t_1–t_3: personal, telephone, or e-mail monitoring every 15 days according to the monitoring questionnaire (MQ).
- V2, "the end of the study visit", 60 days after V1: the MQ, MADRS, DASS, WHOQOL-BREF completion, BP, BMI, WC measurements, blood collection.

4. 0utcome measures

Delta (Δ) was defined as the difference between post-intervention (V2) and pre-intervention (V1) values. %delta (%Δ) was defined as the ratio of Δ to V1 multiplied by 100%.

5. Primary outcome measures

ΔHemoglobin (ΔHGB) is the change in hemoglobin levels over time, reflecting variations in the blood's oxygen-carrying capacity [70].

6. Secondary outcome measures

ΔRed Blood Cells (ΔRBC) is the change in the number of red blood cells, indicating shifts in the body's ability to transport oxygen effectively [71].

ΔHematocrit (ΔHCT) is the change in the percentage of blood volume occupied by red blood cells, often used to assess hydration, anemia, or blood loss [71].

ΔMean Corpuscular Volume (ΔMCV) is the change in the average size of red blood cells, which helps identify different types of anemia or nutritional deficiencies [71].

ΔMean Corpuscular Hemoglobin (ΔMCH) is the change in the average amount of hemoglobin contained in each red blood cell, providing clues to the type and cause of anemia [71].

ΔMean Corpuscular Hemoglobin Concentration (ΔMCHC) is the change in the concentration of hemoglobin within red blood cells, used to evaluate red blood cell health and potential disorders [71].

ΔRed Cell Distribution Width (ΔRDW) is the change in the variation of red blood cell sizes, often associated with anemia and other hematological conditions [72].

7. Tertiary outcome measures

Weight (Wt) is measurement of body mass, typically used to assess overall health and nutritional status [73].

Body Mass Index (BMI) is a ratio of weight to height, calculated to determine whether an individual is underweight, normal weight, overweight, or obese. BMI was computed by dividing weight by the square of height, expressed in kg/m^2. A BMI value of ≥25 indicated overweight ("grade I obesity"), while ≥30 indicated obesity ("grade II obesity") [73].

Waist Circumference (WC) is measurement, which serves as an indicator of central adiposity and risk of metabolic diseases. WC was measured along the midaxillary line, between the lowest rib and the top of the iliac crest [74].

Waist-to-Weight Index (WWI) was calculated as WC divided by the square root of weight (√weight), serves as a novel obesity index that offers advantages over BMI and WC in assessing lean and fat mass [75] .

Waist-to-Height Ratio (WHtR) is comparison of waist circumference to height, used to evaluate the distribution of body fat and potential health risks. WHtR has been identified as more effective than waist circumference (WC) and Body Mass Index (BMI) in detecting cardiometabolic risk factors in both men and women [76].

Blood Pressure (BP) is force of circulating blood on the walls of blood vessels, measured to evaluate cardiovascular health [77].

Fasting Glucose (fGlc) is level of glucose in the blood after a period of fasting, often used to assess risk for diabetes or metabolic disorders [78].

High-Density Lipoprotein Cholesterol (HDL-c) is type of cholesterol known as "good" cholesterol [79].

Non-HDL Cholesterol (non-HDL-c): is total of all cholesterol types other than HDL, including LDL, often linked to cardiovascular risk [80].

Triglycerides (TG) is a form of fat found in the blood that can be used for energy or stored, with high levels often associated with metabolic diseases [81].

Alanine Aminotransferase (ALT) is liver enzyme that helps metabolize amino acids; elevated levels may indicate liver damage or disease [82].

Aspartate Aminotransferase (AST) is liver enzyme used to assess liver health, often elevated alongside ALT in liver disorders [82].

ALT/AST is ratio of ALT to AST, often used to evaluate liver health and differentiate between various liver conditions [83].

Hepatic Steatosis Index (HSI) is non-invasive marker combining clinical and biochemical parameters to estimate the presence of fatty liver disease [84].

C-reactive Protein (CRP) is protein produced in response to inflammation, widely used as a biomarker for systemic inflammation [85].

Neutrophils (NEU) are type of white blood cell critical in the body's first response to infections and injuries [71].

Lymphocytes (LYM) are subtypes of white blood cells involved in adaptive immunity, playing a vital role in defending against infections and abnormal cells [71].

Monocytes (MON) are white blood cells that act as precursors to macrophages and dendritic cells, helping in immune response [71].

Platelets (PLT) are blood components that aggregate to form clots, essential for preventing excessive bleeding [71].

Systemic Immune-Inflammation Index (SII) is composite index incorporating neutrophils, platelets, and lymphocytes to evaluate systemic inflammation and immune activation. SII is calculated as NEU × PLT / LYM and is considered high when it exceeds 600 × 10^9 cells/L [86].

Intestinal Fatty Acid-Binding Protein (I-FABP) is biomarker of gut epithelial damage, often measured to assess intestinal integrity, marker of for intestinal barrier dysfunction, often referred to as "leaky gut" [87].

Tumor Necrosis Factor Alpha (TNF-α) is pro-inflammatory cytokine involved in immune regulation, inflammation, and the pathogenesis of various diseases [88].

Montgomery-Åsberg Depression Rating Scale (MADRS) is clinician-administered tool used to measure the severity of depression symptoms [89].

Depression Anxiety Stress Scales (DASS) is self-reported assessment used to evaluate the emotional states of depression, anxiety, and stress [90].

Quality of Life (QoL) is subjective measure of an individual's overall well-being, encompassing physical, emotional, and social aspects of life [91].

Regarding the **Montgomery-Asberg Depression Rating Scale (MADRS)** is minimum clinically important difference (MCID) was defined as an improvement of at least two points [92], while a clinically meaningful change (CMC) was considered an improvement of at least six points [93]. The treatment response was characterized by a reduction in the initial score of at least 50% [94]. A MADRS score of less than 5 was used to define narrow remission [94]. The MADRS was analyzed using a four-factor model, encompassing sadness, neurovegetative symptoms, detachment, and negative thoughts [94].

Percentage Change in DASS (%ΔDASS) is the overall relative change in the Depression Anxiety Stress Scales score, reflecting shifts in emotional states such as depression, anxiety, and stress [90].

Percentage Change in Depression Subscale of DASS (%ΔD-DASS) is the relative change in the depression subscale of the DASS, specifically assessing variations in depressive symptoms [90].

Percentage Change in Anxiety Subscale of DASS (%ΔA-DASS) is the relative change in the anxiety subscale of the DASS, showing improvements or declines in anxiety levels [90].

Percentage Change in Stress Subscale of DASS (%ΔS-DASS) is the relative change in the stress subscale of the DASS, measuring fluctuations in perceived stress levels [90].

Regarding the **Depression, Anxiety, and Stress Scale (DASS),** the minimum clinically important difference (MCID) was established as an improvement of six points for each subscale, totaling eighteen points for the entire scale, derived from calculations involving clinical outpatient populations [95].

References

68. Harrison, J.E.; Weber, S.; Jakob, R.; Chute, C.G. ICD-11: An International Classification of Diseases for the Twenty-First Century. *BMC Med. Inform. Decis. Mak.* 2021, *21*.
69. Gawlik-Kotelnicka, O.; Strzelecki, D. Probiotics as a Treatment for "Metabolic Depression"? A Rationale for Future Studies. *Pharmaceuticals* 2021, *14*.
70. Płaneta, S.; Bartusik-Aebisher, D.; Aebisher, D. Hemoglobin. In *The Biochemical Guide to Proteins*; Nova Science Publishers, Inc., 2023; pp. 117–121 ISBN 9798886975352.
71. Dean, L. Blood and the Cells It Contains. *Blood Groups Red Cell Antigens* **2005**.
72. Arkew, M.; Gemechu, K.; Haile, K.; Asmerom, H. Red Blood Cell Distribution Width as Novel Biomarker in Cardiovascular Diseases: A Literature Review. *J. Blood Med.* 2022, *13*.
73. Nuttall, F.Q. Body Mass Index: Obesity, BMI, and Health: A Critical Review. *Nutr. Today* 2015, *50*.
74. Thu Tran, N.T.; Blizzard, C.L.; Luong, K.N.; Van Truong, N. Le; Tran, B.Q.; Otahal, P.; Nelson, M.; Magnussen, C.; Gall, S.; Van Bui, T.; et al. The Importance of Waist Circumference and Body Mass Index in Cross-Sectional Relationships with Risk of Cardiovascular Disease in Vietnam.

PLoS One **2018**, *13*, doi:10.1371/journal.pone.0198202.
75. Li, M.; Yu, X.; Zhang, W.; Yin, J.; Zhang, L.; Luo, G.; Liu, Y.; Yang, J. The Association between Weight-Adjusted-Waist Index and Depression: Results from NHANES 2005–2018. *J. Affect. Disord.* **2024**, *347*, doi:10.1016/j.jad.2023.11.073.
76. Louie, J.C.Y.; Wall-Medrano, A. Editorial: Waist-to-Height Ratio Is a Simple Tool for Assessing Central Obesity and Consequent Health Risk. *Front. Nutr.* 2023, *10*.
77. Lewandowska, K.; Wasiliew, S.; Kukfisz, A.; Hofman, M.; Woźniak, P.; Radziemski, A.; Stryczyński, Ł.; Lipski, D.; Tykarski, A.; Uruski, P. Target Blood Pressure Values in Ambulatory Blood Pressure Monitoring. *High Blood Press. Cardiovasc. Prev.* **2023**, *30*, doi:10.1007/s40292-022-00552-3.
78. Lv, K.; Cui, C.; Fan, R.; Zha, X.; Wang, P.; Zhang, J.; Zhang, L.; Ke, J.; Zhao, D.; Cui, Q.; et al. Detection of Diabetic Patients in People with Normal Fasting Glucose Using Machine Learning. *BMC Med.* **2023**, *21*, doi:10.1186/s12916-023-03045-9.
79. Kosmas, C.E.; Martinez, I.; Sourlas, A.; Bouza, K. V.; Campos, F.N.; Torres, V.; Montan, P.D.; Guzman, E. High-Density Lipoprotein (HDL) Functionality and Its Relevance to Atherosclerotic Cardiovascular Disease. *Drugs Context* 2018, *7*.
80. Raja, V.; Aguiar, C.; Alsayed, N.; Chibber, Y.S.; ElBadawi, H.; Ezhov, M.; Hermans, M.P.; Pandey, R.C.; Ray, K.K.; Tokgözoglu, L.; et al. Non-HDL-Cholesterol in Dyslipidemia: Review of the State-of-the-Art Literature and Outlook. *Atherosclerosis* 2023, *383*.
81. Setyawati, R.; Lasroha, M. Overview of HDL, LDL, Triglycerides, and Total Cholesterol in Obese Patients. *Adv. Heal. Sci. Res.* **2021**, *39*.
82. Khan, A.; Ali, S.; Haq, M.U.U.; Sultan, M.; Maqsood, S.; Akhtar, S. Relationship between Alanine and Aspartate Transaminases (ALT and AST) and Fatty Liver on Ultrasound. *Pakistan J. Med. Heal. Sci.* **2021**, *15*, 1610–1613, doi:10.53350/pjmhs211571610.
83. Hall, P.; Cash, J. What Is the Real Function of the Liver 'Function' Tests? *Ulster Med. J.* **2012**, *81*.
84. Priego-Parra, B.A.; Triana-Romero, A.; Martínez-Pérez, G.P.; Reyes-Diaz, S.A.; Ordaz-Alvarez, H.R.; Bernal-Reyes, R.; Icaza-Chávez, M.E.; Martínez-Vázquez, S.E.; Cano-Contreras, A.D.; Vivanco-Cid, H.; et al. Hepatic Steatosis Index (HSI): A Valuable Biomarker in Subjects with Metabolic Dysfunction-Associated Fatty Liver Disease (MAFLD). *Ann. Hepatol.* **2024**, *29*, doi:10.1016/j.aohep.2024.101391.
85. Nehring, S.M.; Goyal, A.; Bansal, P.; Patel, B.C. *C Reactive Protein [Updated 2021 May 10]*; 2021; Vol. 65;.
86. Wang, Q.; Zhu, D. The Prognostic Value of Systemic Immune-Inflammation Index (SII) in Patients after Radical Operation for Carcinoma of Stomach in Gastric Cancer. *J. Gastrointest. Oncol.* **2019**, *10*, 965–978, doi:10.21037/jgo.2019.05.03.
87. Stevens, B.R.; Goel, R.; Seungbum, K.; Richards, E.M.; Holbert, R.C.; Pepine, C.J.; Raizada, M.K. Increased Human Intestinal Barrier Permeability Plasma Biomarkers Zonulin and FABP2 Correlated with Plasma LPS and Altered Gut Microbiome in Anxiety or Depression. *Gut* 2018, *67*.
88. Jang, D.I.; Lee, A.H.; Shin, H.Y.; Song, H.R.; Park, J.H.; Kang, T.B.; Lee, S.R.; Yang, S.H. The Role of Tumor Necrosis Factor Alpha (Tnf-α) in Autoimmune Disease and Current Tnf-α Inhibitors in Therapeutics. *Int. J. Mol. Sci.* 2021, *22*.
89. Carmody, T.J.; Rush, A.J.; Bernstein, I.; Warden, D.; Brannan, S.; Burnham, D.; Woo, A.; Trivedi, M.H. The Montgomery Äsberg and the Hamilton Ratings of Depression: A Comparison of Measures. *Eur. Neuropsychopharmacol.* **2006**, *16*, doi:10.1016/j.euroneuro.2006.04.008.
90. Ali, A.M.; Alkhamees, A.A.; Hori, H.; Kim, Y.; Kunugi, H. The Depression Anxiety Stress Scale 21: Development and Validation of the Depression Anxiety Stress Scale 8-Item in Psychiatric Patients and the General Public for Easier Mental Health Measurement in a Post COVID-19 World. *Int. J. Environ. Res. Public Health* **2021**, *18*, doi:10.3390/ijerph181910142.
91. Phyo, A.Z.Z.; Freak-Poli, R.; Craig, H.; Gasevic, D.; Stocks, N.P.; Gonzalez-Chica, D.A.; Ryan, J. Quality of Life and Mortality in the General Population: A Systematic Review and Meta-

Analysis. *BMC Public Health* **2020**, *20*, doi:10.1186/s12889-020-09639-9.
92. Duru, G.; Fantino, B. The Clinical Relevance of Changes in the Montgomery-Asberg Depression Rating Scale Using the Minimum Clinically Important Difference Approach. *Curr. Med. Res. Opin.* **2008**, *24*, doi:10.1185/030079908X291958.
93. Turkoz, I.; Alphs, L.; Singh, J.; Jamieson, C.; Daly, E.; Shawi, M.; Sheehan, J.J.; Trivedi, M.H.; Rush, A.J. Clinically Meaningful Changes on Depressive Symptom Measures and Patient-Reported Outcomes in Patients with Treatment-Resistant Depression. *Acta Psychiatr. Scand.* **2021**, *143*, doi:10.1111/acps.13260.
94. Quilty, L.C.; Robinson, J.J.; Rolland, J.P.; Fruyt, F. De; Rouillon, F.; Bagby, R.M. The Structure of the Montgomery-Åsberg Depression Rating Scale over the Course of Treatment for Depression. *Int. J. Methods Psychiatr. Res.* **2013**, *22*, doi:10.1002/mpr.1388.
95. Ronk, F.R.; Korman, J.R.; Hooke, G.R.; Page, A.C. Assessing Clinical Significance of Treatment Outcomes Using the Dass-21. *Psychol. Assess.* **2013**, *25*, doi:10.1037/a0033100.
